# Cashmere Blended with Calcium Alginate Fibers: Eco-Friendly Improvement of Flame Retardancy and Maintenance of Hygroscopicity

**DOI:** 10.3390/polym17111497

**Published:** 2025-05-28

**Authors:** Yujie Cai, Zewen Li, Bin Wang, Chao Xu, Xing Tian, Fengyu Quan

**Affiliations:** State Key Laboratory of Bio-Fibers and Eco-Textiles, Institute of Marine Bio-based Materials, College of Materials Science and Engineering, Qingdao University, Qingdao 266071, China; jessicacai1130@163.com (Y.C.); 17660706137@163.com (Z.L.); wangbin20010924@163.com (B.W.); 15633038803@163.com (C.X.)

**Keywords:** calcium alginate fiber, cashmere, flame retardancy, hygroscopicity

## Abstract

As a natural fiber, cashmere is favored for its softness, finesse, and warmth. However, its poor flame-retardant properties seriously affect the safety of cashmere. Current flame-retardant treatments for cashmere tend to lead to heavy metal pollution and significantly reduce wearer comfort. In this work, natural and environmentally friendly calcium alginate fibers were blended with cashmere to obtain blended fibers. The blended fibers exhibited good hygroscopicity and softness. The incorporation of calcium alginate fibers enhanced the flame retardancy of the blends, and the LOI of the blended fibers reached 40.2 without smoldering. It was due to a stable CaO protective layer formed by Ca^2+^ during combustion and the dense carbon layer with the decomposition intermediates of cashmere, which exerted a flame-retardant effect in the condensed phase. This study provided an eco-friendly approach to producing high-quality flame-retardant cashmere products.

## 1. Introduction

Cashmere is a kind of fiber obtained from the refined separation of wool, highly respected for its softness, delicacy, and warmth [1]. It is used widely in high-end clothing and household products, known as Fiber Gem and Fiber Queen [2,3]. However, cashmere has poor flame-retardant properties, with a limiting oxygen index (LOI) of about 25.0%. With the improvement of human living standards, cashmere products frequently appear in public places, such as hotels, conference rooms, and waiting rooms, which are highly required for the flame-retardant performance of the products [4,5,6,7]. The existing cashmere cannot meet the aforementioned needs. Therefore, the flame-retardant modification of cashmere is receiving increasing attention [8].

Like ordinary wool, the chemical method is one of the essential flame-retardant modification methods for cashmere [9,10,11]. Currently, the most widely used method of flame-retardant modification of wool is the Zirpro [12] method, which utilizes a chemical reaction between Ti or Zr complexes and wool to improve the flame-retardant properties. Although it improves the flame retardancy of wool, the K_2_TiF_6_ and K_2_ZrF_6_ used in this method are highly toxic and cause intense irritation on skin contact. In addition, treating wastewater with heavy metal compounds is very complicated. In recent years, researchers have proposed some new flame-retardant methods for wool: Cheng et al. [13] used natural phytic acid and TiO_2_ to construct a new type of organic–inorganic flame-retardant system for wool, which improved the thermal stability and smoke suppression performance of treated wool; Zhang et al. [14] prepared a series of boron-doped silica sols using tetraethyl silicate as the raw material of inorganic precursor and coated them on the surface of wool fabrics, which improved the flame-retardant performance of wool effectively. Although these methods equally improved the flame-retardant properties of wool, they dramatically affected the feel of cashmere products and brought environmental pollution.

Moreover, blending is also an important method to improve the flame retardancy of wool. For example, Flambard et al. [15] blended wool with para-aramid fiber (ArF) to obtain blended fibers with a low heat release rate (HRR) and high thermal stability; Lv et al. [16] blended wool with polyimide (PI) fibers to obtain blended fibers with a limiting oxygen index (LOI) of 31.0%. Although blending has the characteristics of a simple process and no pollution, the extremely low hygroscopicity (moisture regain: ArF = 0.1%, PI = 1.4%) results in a poor body feel and seriously damages the comfort of cashmere products. Therefore, it remains a challenge to improve the flame-retardant properties of cashmere and maintain its hygroscopicity in a simple and environmentally friendly way.

In this work, we used calcium alginate fiber (AF) to prepare cashmere/AF blended fiber by blending and investigated its flame-retardant and hygroscopic properties. As alginic acid is a biopolysaccharide extracted from marine plants with good renewability, biocompatibility, and degradability [17], it has many applications in several fields [18,19]. Meanwhile, the abundant hydrophilic groups on the surface give AF good hygroscopicity [18]. More importantly, AF has excellent intrinsic flame retardancy and low smoke generation [19]. Furthermore, existing studies have shown that blending AF with other fibers will obtain blended fibers with improved flame-retardant properties [19,20,21,22,23]. This work provided a new approach to the eco-friendly manufacture of high-quality, flame-retardant cashmere products.

## 2. Materials and Methods

### 2.1. Materials

Calcium alginate fibers (1.5 dtex, 38 mm) were purchased from Qingdao Yuanhai New Material Technology Co., Ltd. (Qingdao, China). Cashmere (origin: Nei Mongol) was purchased from Zhangjiagang Yangtse Spinning Co., Ltd. (Zhangjiagang, China). Flame-retardant polyester fibers (1.5 dtex, 38 mm, LOI = 30.2 ± 0.4%) Zhejiang Hengchao Chemical Fiber Co., Ltd. (Jiaxing, China). Flame-retardant acrylic fibers (1.3 dtex, 38 mm, LOI = 28.0 ± 0.2%) were purchased from Sinopec Anqing Acrylic Fiber Co., Ltd. (Anqing, China).

### 2.2. Preparation of Cashmere/Calcium Alginate Blended Fibers

Cashmere and calcium alginate fibers were put into a carding machine (SSHFX-3, NanTong SanSi Electromechanical Science & Technology Co., Ltd., Nantong, China) at a mass ratio of 80:20, 60:40, 50:50, 40:60, or 20:80. After mechanical mixing, cashmere/calcium alginate blended fiber bundles were obtained, labeled as C_80_A_20_, C_60_A_40_, C_50_A_50_, C_40_A_60_, and C_20_A_80_. Following the same method, cashmere/polyester blended fiber bundles and cashmere/acrylic blended fiber bundles were obtained, labeled as C_50_P_50_ and C_50_AC_50_, respectively. Additionally, we prepared cashmere bundles and calcium alginate fiber bundles by treating cashmere and calcium alginate fiber in the carding machine for comparison, labeled as Cashmere and AF, respectively.

### 2.3. Characterization

The surface morphology of the samples was analyzed by scanning electron microscopy (SEM, Quanta 250 FEG, FEI NanoPorts, Hillsboro, OR, USA) at an accelerating voltage of 10 kV. Previously, the samples were coated with a conductive layer of platinum. The LOI values of the samples were tested by the HC-2 limited oxygen index instrument (Nanjing Jiangning Analytical Instrument Co., Ltd., Nanjing, China) based on GB/T 2406.3-2022 [24]. The specimen size was 100 × 10 × 4 mm^3^. According to the ISO Standard 5660-1 [25], the flame-retardant properties of the samples were analyzed using a Dual Cone Calorimeter (CONE, 6810, VOUCH, Suzhou, China) at a heat flux of 35 Kw/m^2^. Specimens measuring 100 × 100 × 2 mm^3^ and weighing 10 ± 0.1 g were used. Thermal stability of samples was measured by the thermogravimetric analyzer (TG, TGA2, Mettler Toledo, Greifensee, Switzerland) with a heating rate of 10 °C/min in N_2_ or air at 50 to 800 °C. The sample mass is 10 ± 0.5 mg. The pyrolysis groups in the gas phase were analyzed by a TG-FTIR instrument (TG, TGA2, Mettler Toledo, Switzerland; FTIR, Nicolet Is50, Thermo Fisher, Waltham, MA, USA) in air with a heating rate of 10 °C/min at 50 to 800 °C and a flow rate of 50 Ml/min. The sample mass is 10 ± 0.5 mg. Residue structure and composition were characterized by X-ray photoelectron spectroscopy (XPS, ESCALAB Xi+, Thermo Scientific, Waltham, MA, USA), Raman spectroscopy (RS, DXR2, Thermo Scientific, Waltham, MA, USA), and X-ray diffractometer (XRD, DX2700, Dandong Haoyuan Instrument Co., Ltd., Dandong, China). The moisture regain of the samples was tested based on GB/T 9994-2018 [26]. The sample mass is 100 ± 3 g. The test environment temperature was 20 ± 2 °C, and humidity was 65 ± 4%. The softness of the samples was tested by the Y331LN digital yarn twist tester (Changzhou Dedu precision instruments Co. Ltd., Changzhou, China) based on GB/T 12411-2006 [27]. The sample mass is 100 ± 5 mg. The test environment temperature was 20 ± 2 °C, and humidity was 65 ± 3%.

## 3. Results

### 3.1. Structural Characterizations

**Figure 1:**
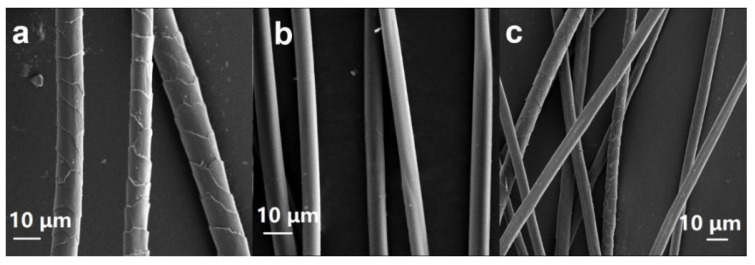
SEM images of (**a**) cashmere, (**b**) AF, and (**c**) C_50_A_50_ Samples.

Using SEM, the micro-morphology of the samples was observed. As shown in Figure 1, the micro-morphology of AF and cashmere fibers differed significantly. The surface of AF was relatively smooth, and there were some grooves. The cashmere fibers had evenly distributed scales on the surface, which is the typical morphology of animal fibers. In C_50_A_50_, the two different micro-morphologies of fibers distribute equally, indicating that the cashmere and AFs were well mixed.

### 3.2. Flame Properties

**Figure 2:**
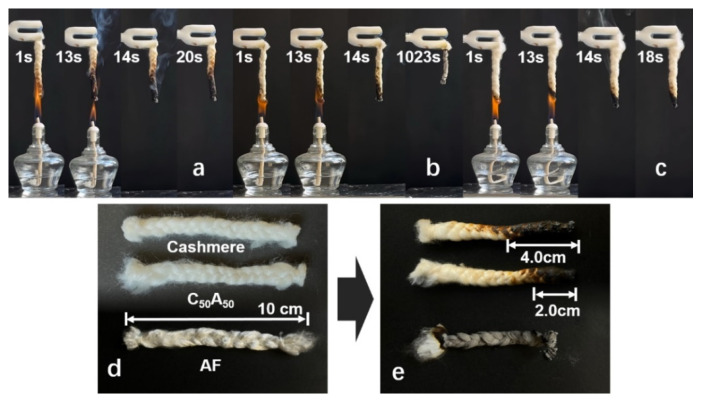
Combustion of samples in the air. (**a**) Cashmere, (**b**) AF, (**c**) C_50_A_50_, (**d**) samples before combustion, and (**e**) samples after combustion.

The flame retardancy of the blended fibers was characterized by LOI and combustion tests, as presented in Figure 2 and Table 1. The results showed a significant increase in LOI values with a higher content of the AF, while the damage length caused by burning decreased gradually. This improvement in flame retardancy was due to the high LOI value of AF (45.0 ± 0.4) [28,29,30]. Notably, although swiftly extinguished, the AF samples underwent prolonged smoldering until destroyed, consistent with some previous reports. This was due to the fact that calcium alginate had a low initial decomposition temperature. When the mass ratio of AF to cashmere exceeded 1:1, all samples displayed smoldering. However, when the cashmere content was higher, the samples exhibited reduced afterglow times and shorter damage lengths, indicating that cashmere diminishes the smoldering tendency of alginate fibers. In particular, the C_50_A_50_ exhibited the shortest afterglow time and damage length, demonstrating the most effective flame-retardant properties. Therefore, future research focused on the C_50_A_50_.

**Figure 3:**
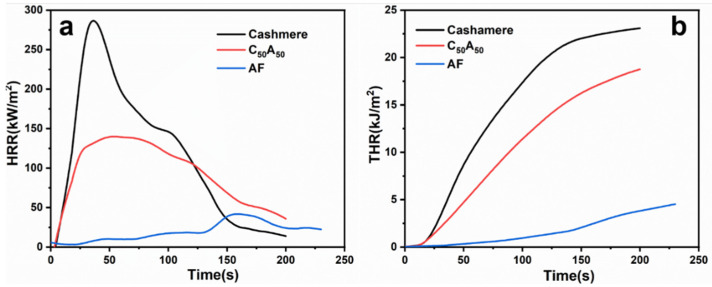
(**a**) HRR and (**b**) THR for the samples.

The flame-retardant properties of samples were evaluated by cone calorimeter tests, as illustrated in Figure 3 and Table 2. The time to ignition (TTI) of cashmere was about 8 s, followed by rapid combustion and significant heat release. It indicated that the combustion process of cashmere is more dramatic and poses a dangerous fire hazard, even though it could self-extinguish. In contrast, AF was difficult to ignite and exhibited a lower peak heat release rate (pHRR), total heat release (THR), and total smoke production (TSP). Although C_50_A_50_’s TTI was not significantly improved compared to cashmere, its pHRR, THR, and TSP were lower than those of cashmere samples. Besides that, the effective heat of combustion (EHC) of C_50_A_50_ was lower than that of cashmere, suggesting reduced combustion and heat generation from cashmere volatile pyrolysis products. Further, the fire growth rate (FIGRA) [31] of cashmere decreased from 9.2 to 5.4 after blending, demonstrating that adding AF reduced the fire hazard of cashmere.

### 3.3. Thermal Stability

**Figure 4:**
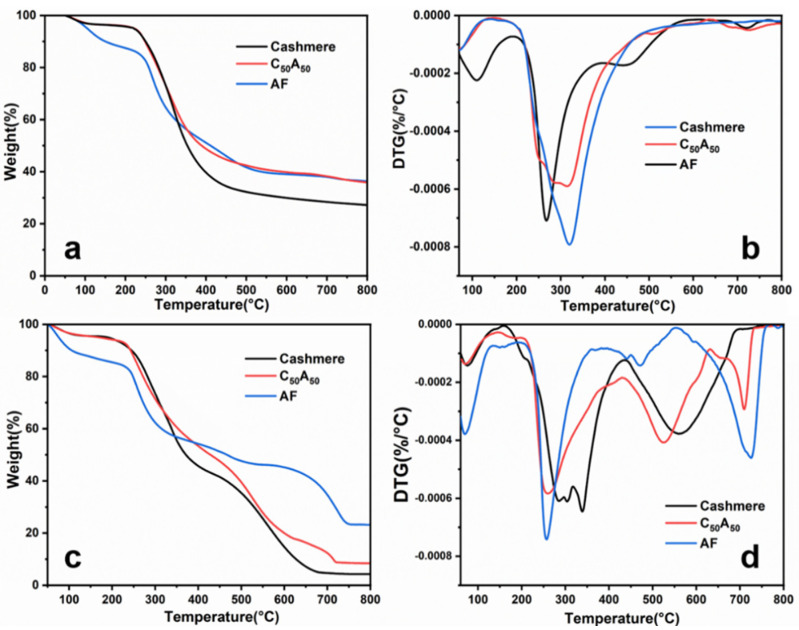
TG (**a**,**c**) and DTG (**b**,**d**) curves for samples in N_2_ and air.

The thermal stability of the samples was analyzed using TGA, and Figure 4 shows the results. Table 3 and Table 4 present the relevant details.

From Figure 4a,b, there were four stages of weight loss in AF in N_2_: (1) removal of bound water from the fiber (50–195 °C) [32], (2) decarboxylation and glycosidic bond cleavage (195–395 °C), (3) reaction of intermediates to form charcoal and CaCO_3_ (395–586 °C) [33], and (4) the decomposition of CaCO_3_ to CaO (586–752 °C) [34,35]. The weight loss of cashmere in N_2_ had two main stages: (1) removal of bound water (50–170 °C) and (2) peptide bond breaking and intermediate product decomposition (130–545 °C) [36,37]. The above decomposition processes could also be found in C_50_A_50_′s similar temperature range with corresponding weight loss stages, suggesting that cashmere and AF in the blended fibers decompose independently. However, the residual carbon rate of the C_50_A_50_ was close to that of AF, attributed to the decomposition of cashmere into intermediates during the low-temperature phase, which promoted the formation of a stable carbon layer in the alginate fibers.

As shown in Figure 4c,d, in air, AF exhibited a similar weight loss behavior as in N_2_, but the reaction of oxygen with the carbon layer generates heat that accelerates CaCO_3_ decomposition, resulting in a significant weight loss in the fourth stage. There was no noticeable weight loss after 760 °C. In contrast to the decomposition process in N_2_, cashmere in air showed a third stage of weight loss (450–700 °C) with a dramatic decrease in residues. It was attributed to the reaction between oxygen and protein intermediates, leading to further decomposition of the cashmere [38]. The first two decomposition stages of C_50_A_50_ in air were similar to that of AF, whereas the decomposition stages after 430 °C corresponded to the third stage of weight loss of cashmere and the fourth stage of decomposition of AF, respectively, with a higher amount of residue than that of cashmere. Unlike cashmere and AF, the last two decomposition stages of C_50_A_50_ showed decreased ending temperature, caused by the fact that Ca^2+^ promoted the reaction between proteolytic intermediates and oxygen [32]. At the same time, CaCO_3_ absorbed the heat released by the reaction and decomposed more rapidly into CaO [39].

### 3.4. TG-FTIR Analysis

**Figure 5:**
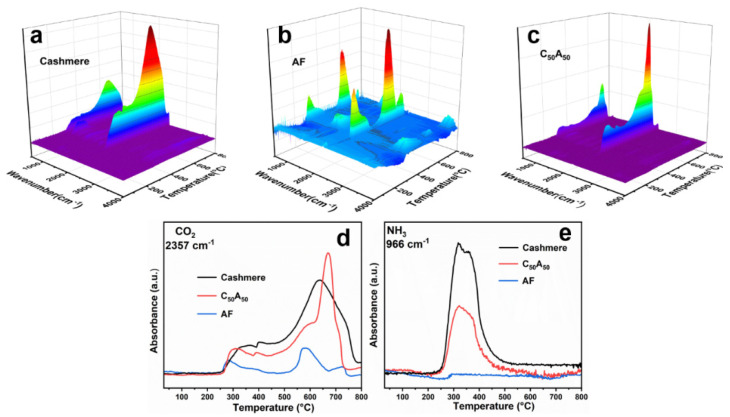
(**a**–**c**) Three-dimensional diagrams of TG-FTIR for samples; (**d**,**e**) 966 cm^−1^ and 2357 cm^−1^ absorbance for samples at different temperatures.

We investigated the gas-phase products of thermal degradation of cashmere, AF, and C_50_A_50_ by TG-FTIR, as shown in Figure 5. The results showed that the vibrational absorption peaks of NH_3_ (966 cm^−1^) existed in the gas-phase products of both cashmere and C_50_A_50_ and appeared at similar temperatures, indicating that the AF did not change the initial decomposition of proteins in cashmere [40,41]. However, comparing the position of the CO_2_ absorption peak (2357 cm^−1^) in AF and C_50_A_50_ indicates that CaCO_3_ in the composite fiber completes its decomposition faster, attributed to the heat released from the protein intermediates’ decomposition accelerating the decomposition of CaCO_3_ into CaO.

### 3.5. Char Residues Analysis

**Figure 6:**
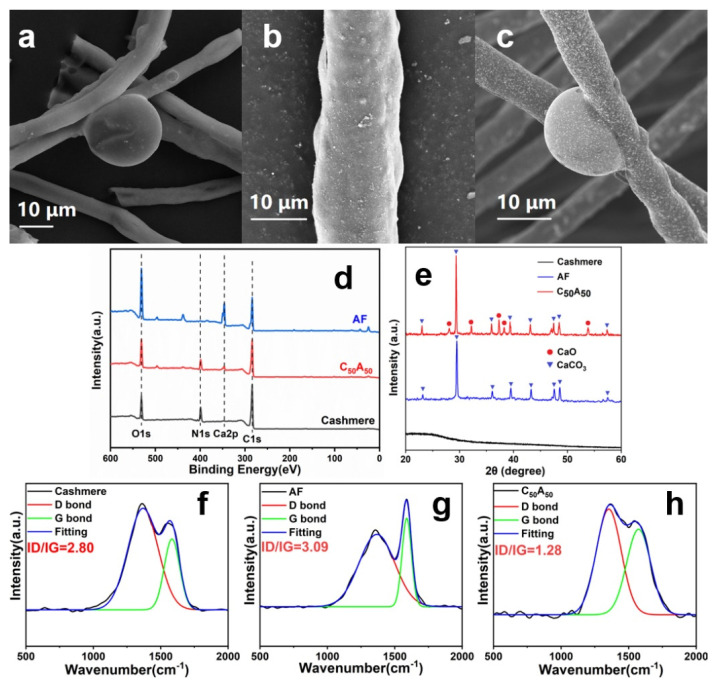
SEM images of (**a**) cashmere, (**b**) AF, (**c**) C_50_A_50_, (**d**) XPS for the residues, (**e**) XRD for the residues, and (**f**–**h**) RS for the residues.

The microscopic morphology of the sample residue after combustion was investigated by SEM, as presented in Figure 6a–c. It showed that the surface scale-like morphology disappeared in the cashmere residue, and bubbles were caused by the gas generated in the combustion. There were many particles on the surface of the AF’s residue. The residue of C_50_A_50_ was not only bubbled but also covered with dense particles on the surface.

In addition, we analyzed the composition of the post-combustion residue samples using XPS and XRD, as shown in Figure 6e. The results showed undecomposed N in the cashmere residue. Still, Ca and crystal diffraction peaks were absent. In contrast, Ca appeared in the AF residue, whose diffraction peak corresponded to the CaCO_3_ characteristic line. There were N and Ca in the residue of C_50_A_50_, and the more complex diffraction peaks corresponding to CaO and CaCO_3_ suggested that CaCO_3_ in the blended fibers is more likely to decompose and generate CaO.

Moreover, we used RS to characterize the structure of the residual carbon, as shown in Figure 6f–h. The G and D peaks in the Raman spectroscopy reflect the graphitic and defective structures, respectively. The smaller the ID/IG value, the higher the graphitized degree of the sample and the better its thermal stability [42]. The fitted data showed that the ID/IG values of the residues in cashmere and AF were 2.80 and 3.09, respectively. While the C_50_A_50_ was only 1.28, which indicated that the ring-forming effect of the cashmere’s decomposition intermediates and calcium ions during the combustion process of the co-blended fibers effectively improved the integrity of the graphite structure in the residual char layer and thus improved the flame-retardant effect.

### 3.6. Flame Retardancy Mechanism of Blended Fiber

The combustion tests confirmed that the AF effectively enhanced the flame-retardant performance of cashmere fiber, while cashmere also inhibited the smoldering of AF. Cone calorimetry showed that the heat generation of C_50_A_50_ was significantly reduced, further reducing the secondary hazards caused by combustion. TGA showed that calcium ions in calcium alginate promote rapid reaction between proteolytic intermediates and oxygen, generating a graphitized carbon layer with a more complete structure. At the same time, the generated heat accelerated the degradation of CaCO_3_ into a protective layer of CaO. Considering these results, the primary mechanism by which calcium alginate fibers improve the flame retardancy of cashmere is through the rapid formation of a stable and complete charcoal layer and a CaO protective layer. This action is achieved by Ca^2+^ catalyzing the formation of a graphitized char layer from the decomposition intermediates of cashmere and accelerating the rapid decomposition and heat absorption of CaCO_3_, acting in the condensed phase.

### 3.7. Hygroscopicity

**Figure 7:**
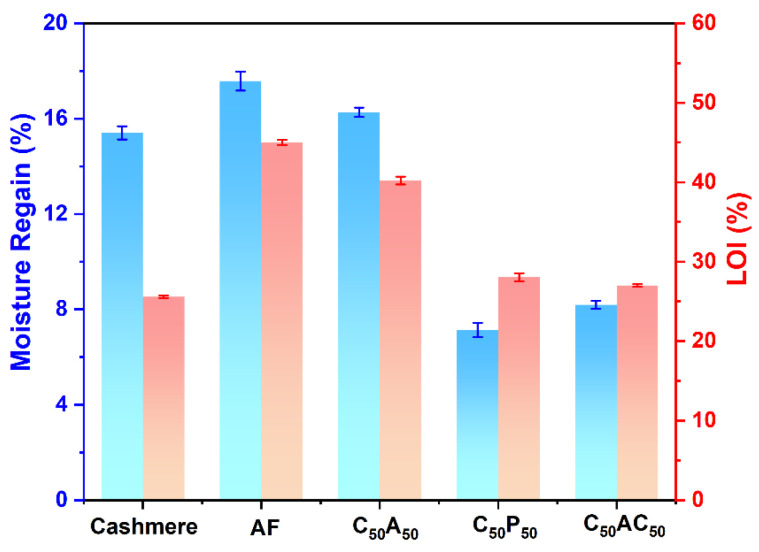
Moisture regain and LOI of samples.

Hygroscopicity is an essential factor in determining the comfort level of fibers [43,44]. The moisture regain was used to characterize the hygroscopicity of the samples, as shown in Figure 7. It revealed that benefiting from a large number of hydrophilic groups on the surface, both cashmere and AF showed very high moisture regain of 15.40% and 17.57%, respectively, which is one of the most important reasons for the comfort of both fibers. However, when blended cashmere with flame-retardant polyester or flame-retardant acrylic, the flame-retardant properties of the blended fibers increased slightly, but the moisture regain decreased significantly, greatly affecting the comfort. On the contrary, even with an LOI of about 40%, C_50_A_50_ still showed a moisture regain of 16.26%, which provided both safety and a better body feeling.

In addition, the degree of softness significantly impacts the comfort of the fiber. The softness of the samples was evaluated by the breaking twist number, as shown in Table 5. The results show that at similar fiber fineness, the average breaking twist number of AF and cashmere reached 10 twists/10 cm, suggesting they have comparable softness. Therefore, the average breaking twist number of C_50_A_50_ was 10 ± 1 twists/10 cm, consistent with that of cashmere fiber, which showed good softness. In contrast, the average breaking twist number was reduced in cashmere blended with either flame-retardant polyester or acrylic fibers, indicating a deterioration in softness. The decrease was less pronounced in the C_50_AC_50_ blend, attributed to the lower fineness of the flame-retardant acrylic fiber (1.3 dtex).

## 4. Conclusions

In this work, we successfully enhanced the flame-retardant properties of the blended fibers while maintaining good hygroscopic properties and softness by mixing the environmentally friendly calcium alginate fibers with cashmere. The LOI of the blended fibers could reach 40.2 without smoldering, the HRR, THR, TSP, and EHC were significantly reduced, and the flame-retardant effect improved considerably. This improvement was attributed to the stabilized CaO protective layer generated by Ca^2+^ in the calcium alginate fibers and the denser charcoal layer generated by the reaction between Ca^2+^ and the decomposition intermediates of cashmere, which played a flame-retardant effect in the condensed phase. The moisture regain of C_50_A_50_ was up to 16.26%, and the breaking twist number was 10 ± 1 twists/10 cm, similar to that of cashmere. It was significantly better than cashmere/flame-retardant polyester or cashmere/flame-retardant acrylic blended fiber and demonstrated excellent comfort. This study provided an eco-friendly path to develop high-quality flame-retardant cashmere products.

## Figures and Tables

**Table 1:** LOI and combustion test data.

Samples	LOI (%)	Afterflame Time (s)	Afterglow Time (s)	Damage Length (mm)
Cashmere	25.6 ± 0.2	7	0	40
C_80_A_20_	27.3 ± 0.3	6	0	37
C_60_A_40_	33.4 ± 0.2	6	0	28
C_50_A_50_	40.2 ± 0.6	5	0	20
C_40_A_60_	40.8 ± 0.2	0	502	85
C_20_A_80_	43.4 ± 0.4	0	1033	100
AF	45.0 ± 0.4	0	1010	100

**Table 2:** CONE data of the samples.

Samples	p-HRR	Tp-HRR	TTI	THR	TSP	FIGRA
	(kW/m^2^)	(s)	(s)	(MJ/m^2^)	(m^2^)	(kW/m^2^ s)
Cashmere	289.5 ± 15.8	36 ± 2	8 ± 1	23.1 ± 0.8	0.79 ± 0.13	9.2
C_50_A_50_	141.1 ± 9.0	46 ± 1	11 ± 1	18.8 ± 0.5	0.27 ± 0.06	5.4
AF	42.6 ± 2.5	154 ± 5	138 ± 8	4.5 ± 0.2	0.08 ± 0.02	0.3

**Table 3:** Data obtained from TG and DTG in N_2_.

Samples	T_1max_ (°C)	T_2max_ (°C)	T_3max_ (°C)	T_4max_ (°C)	Residues (%)
Cashmere	70.2	319.4	—	—	27.18
C_50_A_50_	72.0	314.8	505.5	733.3	35.87
AF	109.9	267.1	454.4	724.9	36.32

**Table 4:** Data obtained from TG and DTG in air.

Samples	T_1max_ (°C)	T_2max_ (°C)	T_3max_ (°C)	T_4max_ (°C)	Residues (%)
Cashmere	70.5	340.3	561.7	—	4.23
C_50_A_50_	71.2	258.6	525.5	709.7	8.38
AF	78.7	256.8	472.8	725.6	23.17

**Table 5:** Softness for the samples.

Sample	Cashmere	AF	C_50_A_50_	C_50_P_50_	C_50_AC_50_
Breaking twist number (twists/10 cm)	10 ± 2	10 ± 1	10 ± 1	7 ± 1	9 ± 2

## Data Availability

Data are contained within the article.
